# A novel approach to T-cell receptor beta chain (TCRB) repertoire encoding using lossless string compression

**DOI:** 10.1093/bioinformatics/btad426

**Published:** 2023-07-07

**Authors:** Thomas Konstantinovsky, Gur Yaari

**Affiliations:** Faculty of Engineering, Bar Ilan University, Ramat Gan 5290002, Israel; Bar Ilan Institute of Nanotechnology and Advanced Materials, Bar Ilan University, Ramat Gan 5290002, Israel; Faculty of Engineering, Bar Ilan University, Ramat Gan 5290002, Israel; Bar Ilan Institute of Nanotechnology and Advanced Materials, Bar Ilan University, Ramat Gan 5290002, Israel

## Abstract

**Motivation:**

T-cell receptor beta chain (TCRB) repertoires are crucial for understanding immune responses. However, their high diversity and complexity present significant challenges in representation and analysis. The main motivation of this study is to develop a unified and compact representation of a TCRB repertoire that can efficiently capture its inherent complexity and diversity and allow for direct inference.

**Results:**

We introduce a novel approach to TCRB repertoire encoding and analysis, leveraging the Lempel-Ziv 76 algorithm. This approach allows us to create a graph-like model, identify-specific sequence features, and produce a new encoding approach for an individual’s repertoire. The proposed representation enables various applications, including generation probability inference, informative feature vector derivation, sequence generation, a new measure for diversity estimation, and a new sequence centrality measure. The approach was applied to four large-scale public TCRB sequencing datasets, demonstrating its potential for a wide range of applications in big biological sequencing data.

**Availability and implementation:**

Python package for implementation is available https://github.com/MuteJester/LZGraphs.

## 1 Introduction

Living in an environment where pathogens are a constant threat, our adaptive immune system has little to no time to rest, constantly trying to recognize pathogens by binding T- and B-cell-specific receptors to their antigens. In order to produce a proper receptor that could bind to a specific antigen, our body leverages the stochastic V(D)J recombination process, which accounts for the high receptor variability seen in individuals (Janeway and Medzhitov 2002). Understanding the fine details of the mechanism behind the V(D)J recombination and how they affect the dynamics and variability of our adaptive immune system is one of the fundamental open questions in immunology. A proper methodology for a compact representation of an entire repertoire, which encapsulates as much information as possible, is of crucial need (Six *et al.* 2013). Such a representation will provide a potent and convenient way to analyze and infer the different attributes that compose an individual repertoire. While it is a well-established fact that the V(D)J recombination process is highly nondeterministic (Hozumi and Tonegawa 1976, Janeway and Medzhitov 2002, Chi *et al.* 2020), numerous models were suggested to capture recombination statistical properties (Murugan *et al.* 2012, Ralph and Matsen 2016, Marcou *et al.* 2018, Sethna *et al.* 2019, 2020). Few methods were proposed aiming to convert the large number of sequences in an individual repertoire into a single vector representation (Ostmeyer *et al.* 2017, Priel *et al.* 2018, Widrich *et al.* 2020, Ostrovsky-Berman *et al.* 2021, Zhang *et al.* 2021, Chen *et al.* 2022), that can be used for machine learning classification tasks (Greiff *et al.* 2020, Pavlović *et al.* 2021, Shemesh *et al.* 2021, Safra *et al.* 2022, 2023, Zaslavsky *et al.* 2023). Many applications revolving around the storage and processing of data produced by high-throughput processes leverage various compression algorithms (Zhu *et al.* 2015, Numanagić *et al.* 2016, Ginart *et al.* 2018) to apply relevant producers in an efficient manner. These compression algorithms are implemented via different methodologies offering a spectrum of compressed formats and are commonly separated into two groups. Compression algorithms can be classified into two categories: lossless and lossy. Lossless compression guarantees an exact reconstruction of the original file, while lossy compression produces a decompressed file that closely approximates the original data but may not be identical. Lossy algorithms generally achieve higher compression ratios, while lossless algorithms ensure an exact representation of the original data at the cost of lower compression ratios (Popitsch and von Haeseler 2013). Lossless compression algorithms play a crucial role in a wide range of domains, including but not limited to those highlighted in studies such as Bashashati *et al.* (2007), Anderson and Wisneski (2008), Wu *et al.* (2005), and Hu *et al.* (2006). These well-established algorithms are designed to reduce the size of compressed files while ensuring the complete reconstruction of the original data. They achieve this by employing token-based mappings, a technique pioneered by foundational works such as Huffman (1952), Lempel and Ziv (1976), Burrows and Wheeler (1994), Welch (1984), Deutsch (1996), and Horita *et al.* (2019). By effectively representing recurring patterns or subpatterns within the data, these algorithms facilitate efficient storage and transmission. Yet, to the best of our knowledge, there are few published studies attempting to combine these concepts from information theory with data modeling or analysis (Gusev *et al.* 1999, Otu and Sayood 2003). Moreover, in the context of immune repertoires, there are no published studies explicitly exploring the utilization of various lossless data compression algorithms as feature extractors.

**Figure 1.**
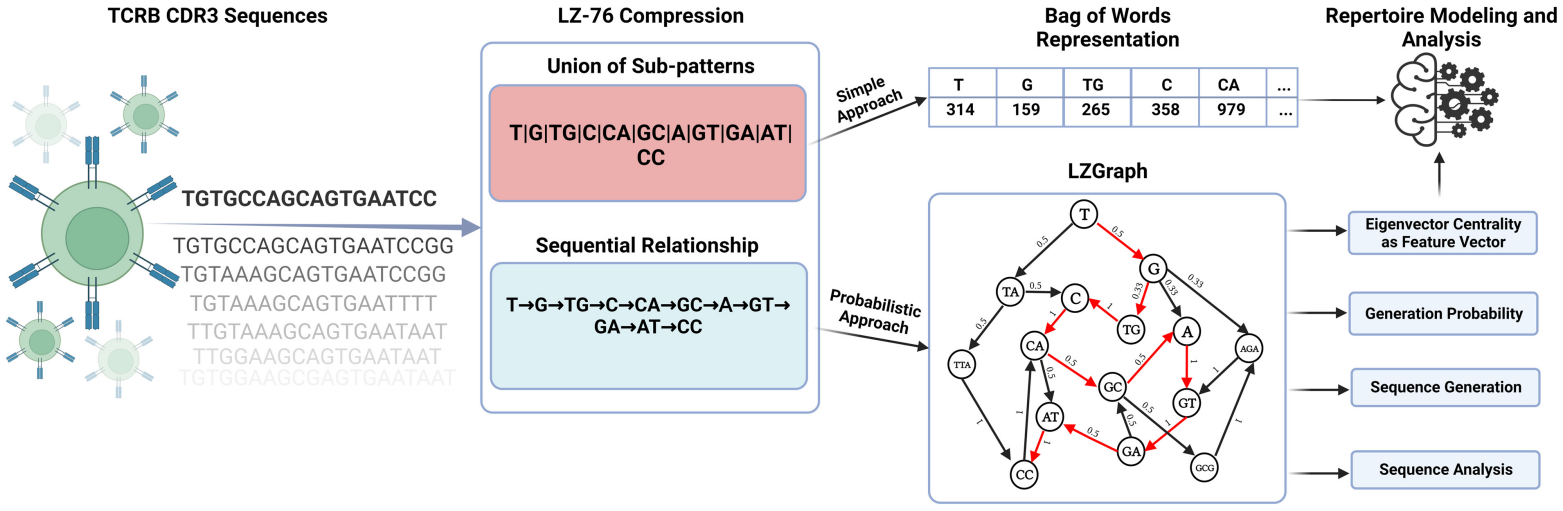
Illustration of the proposed methodology flow, from sequence to model. The diagram showcases the sequential transition from T-cell receptors and their corresponding CDR3 sequences on the left, to the proposed representations and various downstream tasks on the right (created with BioRender.com).

An intriguing question arises regarding the potential of utilizing a compressed representation of a repertoire, along with the accompanying token dictionary, to develop a statistical model that effectively captures the underlying dynamics of the repertoire. Here, we introduce a novel methodology for encoding the CDR3 regions of the T-cell receptor beta chain (TCRB) repertoire into a comprehensive graph-based representation (Fig. 1), utilizing the Lempel-Ziv subpattern extraction algorithm (LZ-76) (Lempel and Ziv 1976). The LZ-76 algorithm operates as a lossless technique that employs a sliding window to identify repeated patterns within the input data. By replacing these recurring patterns with references to previous occurrences in the data, the algorithm produces a compressed output without any loss of information. The encoding approach we propose allows for in-depth repertoire analysis, feature extraction for statistical modeling, synthetic sequence generation, generation probability inference, and a new measure for diversity estimation. We elaborate and demonstrate how, given a repertoire, the resulting model captures its inner dynamics, allowing us not only to classify cohorts of individuals based on their repertoires, but also to generate new synthetic sequences and infer generation probability (${\text{LZP}}_{\text{gen}}$) for each sequence. The resulting repertoire graph model provides researchers with a novel approach to TCRB repertoire encoding. The presented approach has major advantages over existing state of the art models, by not relying on a preliminary, typically noisy, sequence annotation steps, and by being more efficient computationally.

## 2 Materials and methods

### 2.1 Data

The datasets used in this work are composed of TCR beta chain CDR3 sequences. They are summarized in Table 1.

**Table 1.** A summary of the datasets used throughout this paper.

Dataset abbreviation	Condition	Number of repertoires	Reference
DS1	HIV	192	Towlerton *et al.*(2022)
DS2	HIV	39	Simonetti *et al.*(2021)
DS3	CMV and controls	785	Emerson *et al.*(2017)
DS4	COVID	950	Nolan *et al.*(2020)

The development and analysis of the methods were performed on productive CDR3 sequences in both nucleotide and amino acid scopes. TRBV and TRBJ gene annotations were taken for each sequence as provided via “ImmunoSeq Access” (Biotechnologies 2022). No further preprocessing was applied to the repertoire data (Fig. 1).

### 2.2 LZ-76

**Figure 2.**
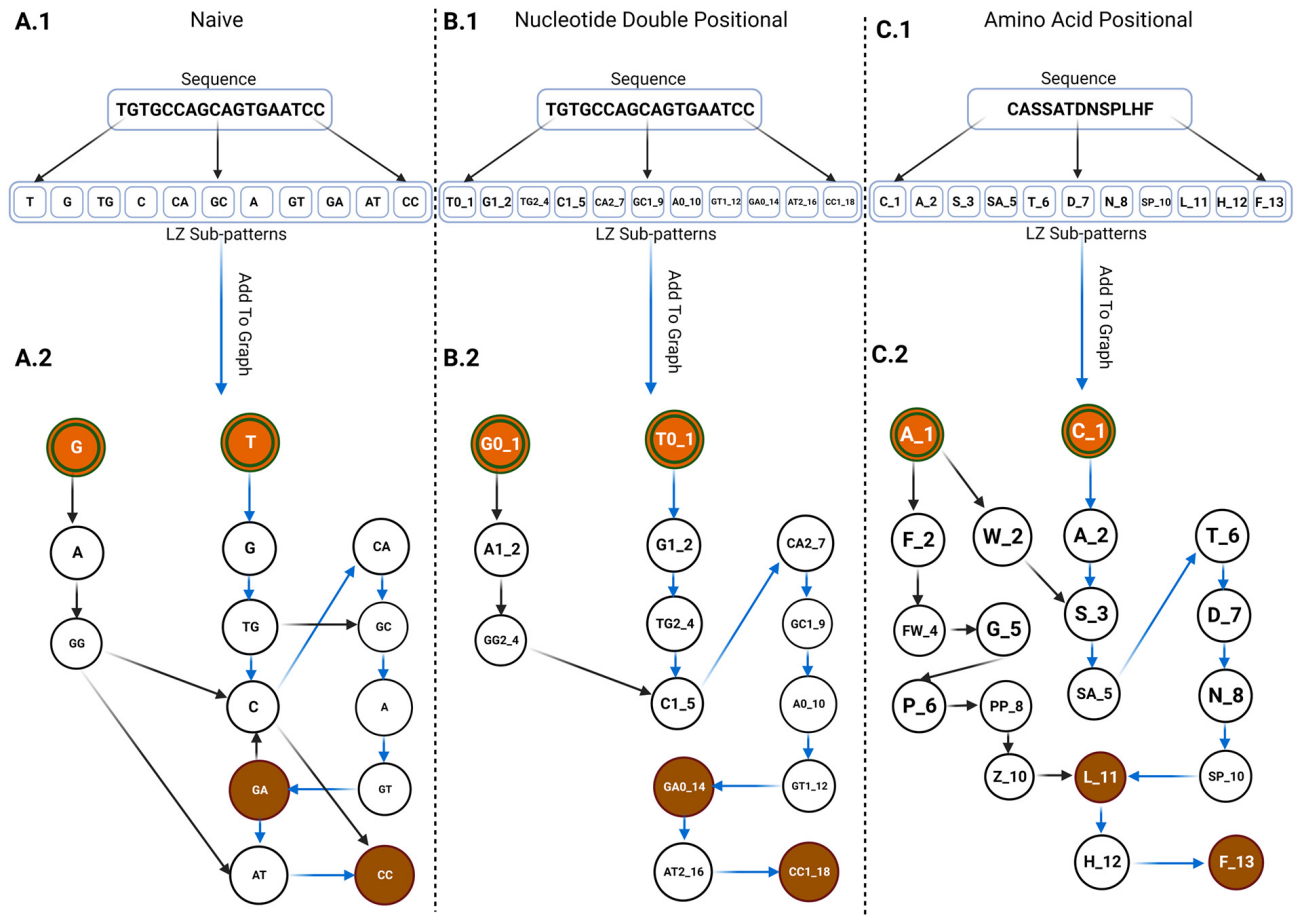
Sequence-to-graph transitions: an illustrative example. In all panels, double circled orange nodes represent the initial states of the graph, while brown nodes indicate terminal states. The blue arrows represent the newly added/updated edges derived from the sequence in Panels X.1 and incorporated into the graph shown in Panels X.2. (A.1) demonstrates the LZ-76 decomposition of a sequence into subpatterns in the “Naive” graph setting. (A.2) showcases the insertion of LZ-76 subpatterns extracted in (A.1) into a graph structure. (B.1) illustrates an example of the “Nucleotide Double Positional” graph node encoding scheme, respecting the imposed Directed Acyclic Graph (DAG) constraint. Nodes are differentiated by the reading frame start position and the position along a sequence. (B.2) displays the insertion of subpatterns extracted in (B.1) into the graph structure. (C.1) showcases an example of the “Amino Acid Positional” graph node encoding scheme, also respecting the DAG constraint. Nodes are differentiated by the reading frame start position and the position along a sequence. (C.2) demonstrates the insertion of subpatterns extracted in (C.1) into the graph structure. (Created with BioRender.com).

The Lempel-Ziv algorithm, initially introduced in the LZ-76 paper, presents a method for extracting unique subpatterns from a given sequence of bits, which is commonly referred to as the “Lempel Ziv Complexity” of the sequence. This algorithm formed the foundation for subsequent developments, such as the compression algorithm presented in the LZ-77 paper. In the LZ-77 approach, an index was assigned to each subpattern, enabling the encoding and compression of a sequence of bits based on these subpatterns. In our study, we applied the fundamental form of the Lempel-Ziv algorithm, as described in the literature (Lempel and Ziv 1976, Ziv and Lempel 1977), to tokenize the input sequences and construct dictionaries of unique subpatterns (Fig. 2). Since there is no inherent ordering of CDR3 sequences in a repertoire, we derived a separate dictionary of subpatterns for each sequence, resulting in *N* dictionaries corresponding to the repertoire depth. The subpattern extraction method we use throughout this paper is formally defined in Algorithm 1.

Algorithm 1 Lempel-Ziv Decomposition

1: **function**  LempelZivDecomposition(sequence: str)

2:   sub_strings←{}

3:   n←length of sequence

4:   ind←0  ▹ Sliding Window Start Position

5:   inc←1  ▹ Sliding Window Size

6:   **while True do**

7:     **if**  ind+inc>n  **then**

8:         **break**

9:       **end if**

10:       **if**  ind+inc=n  **and**  sequence[ind:ind+inc]∉sub_strings  **then**

11:         sub_str←sequence[ind:ind+inc]

12:         sub_strings←sub_strings∪{sub_str}

13:         **break**

14:       **else**

15: sub_str←sequence[ind:ind+inc]

16:       **end if**

17:       **if**  sub_str∈sub_strings  **then**

18:         inc←inc+1

19:       **else**

20:         sub_strings←sub_strings∪{sub_str}

21:         ind←ind+inc

22:         inc←1

23:       **end if**

24:     **end while**

25:   **return**  sub_strings

26: **end function**

A common way to understand the use case of LZ-76 is by considering it as an approximation method for estimating the value of the theoretical Kolmogorov complexity, which is the length of the shortest computer program that is able to reproduce a given pattern (Li and Vitányi 1997). LZ-76 is also an efficient estimator of the entropy rate, which measures how many bits of innovation are introduced by each observation (Aboy *et al.* 2006). Naturally, the more complex a sequence is, the higher the entropy, the more resources will be needed to recreate that sequence, i.e. a higher Kolmogorov complexity.

Due to the greedy nature of LZ-76 algorithm, each time a new subpattern is observed it is added to the aggregated vocabulary. A special case has to be addressed when the last observed sequence of symbols is already a subpattern in our dictionary. Our solution consists of appending those symbols to the last observed subpattern.

### 2.3 LZ-76 bag-of-words representation

One of the proposed approaches for representing TCR repertoires is through a bag-of-words (BOW) framework. This involves constructing a frequency table, known as the BOW feature vector, which captures the occurrence of unique subpatterns derived from all observed sequences within the repertoire. The BOW representation provides an overview of the repertoire composition based on LZ-76 subpatterns. However, this representation overlooks the order and sequential relationships among subpatterns. To address this limitation and effectively capture both the structural patterns and the sequential dependencies within the repertoire, a graph data structure was employed.

### 2.4 Graph-based representation (LZGraph)

The nodes of an LZGraph represent the collection of distinct subpatterns, while the edges of the graph are established by connecting consecutive subpatterns. It is important to note that the order in which the sequences are read does not affect the final model. The graph construction process occurs as the sequences are processed individually, irrespective of their order in the repertoire. The weights assigned to each edge in the graph are proportional to the empirical transition events between the connected nodes, normalized by the out-degree of each node. Optionally, an additional layer of information can be added to each edge documenting the relative V and J gene usage of the sequences contributing to this edge.

Formally the LZGraph of repertoire *x* is defined as the set triplet (V,E,W) that stands for vertices (aka nodes), edges, and weights, respectively. ∀i∈{1..N}. $deg^{-}(v_{i})$ indicates the out-degree of node $v_{i}$. Two additional sets we define are the set of all initial states (*Init*), and the set of all terminal states (*Term*).


(1) $$\begin{array}{c} V=\{v_{i}\} \end{array}.$$

(2) $$\begin{array}{c} E=\{e_{ij}\;|\;(v_{i};v_{j})\;\text{if}\;v_{j}\;\text{is observed following}\;v_{i}\} \end{array}.$$

(3) $$\begin{array}{c} W=\left\{\;w_{ij}\;|\;\frac{\#\text{of observed transitions between}\;v_{i}\;\text{and}\;v_{j}}{{\text{deg}}^{-}(v_{i})}\right\} \end{array}.$$

We present three main graph types: “naïve,” “Nucleotide Double Positional,” and “Amino Acid Positional” graphs. The “Naive” graph represents nodes consisting of LZ-76 nucleic acid subpatterns only, without any additional positional information. In the “Nucleotide Double Positional” graph, the LZ-76 nucleic acid subpattern nodes are suffixed with two pieces of information: the starting position within the reading frame and the absolute location in the sequence. Generally, each node is structured as {LZ-76 sub-pattern}{modulo 3 position (reading frame)}_{absolute position}. Lastly, the nodes in the “Amino Acid Positional” graph are the LZ-76 subpatterns of the CDR3 amino acid sequence, with an appended suffix indicating the start position of the subpattern within the sequence. For example, a node like LF_7 represents the subpattern “LF” (Leucine-Phenylalanine), starting at the seventh position in the CDR3 amino acid sequence.

The insertion of positional components to each subpattern in the graphs enforces the resulting LZGraph to be a directed a-cyclical graph (DAG). A DAG graph ensures that there is a constant flow from the root node (a node belonging to the *Init* set defined above) to the terminal node (a node belonging to the *Term* set defined above), avoiding any cycles which in a “Naive” graph occur naturally. The incorporation of positional information enhances the connectivity and coherence of the LZGraphs, as each node can be precisely linked to a specific position in the CDR3 sequence. This augmentation of positional information not only promotes greater interoperability within the graph but also significantly improves its suitability for downstream tasks, such as sequence generation and probability inference. By capturing the accurate positioning of subpatterns, the graph attains enhanced fidelity, leading to more precise and reliable outcomes in these subsequent analyses.

#### 2.4.1 Generation probability (${\text{LZP}}_{\text{gen}}$)

$P_{\text{gen}}$ is a measure utilized to estimate the generation probability of a specific sequence (Marcou *et al.* 2018). Various generation models can be employed to calculate this probability. Notably, existing models for generation probability estimation rely on mechanistic modeling of the underlying biological process. In contrast, the LZ-76 ${\text{LZP}}_{\text{gen}}$s derived from the graph types depicted in Fig. 2 are solely based on the structural characteristics of the sequence itself. This distinctive attribute enables the estimation of generation probability without necessitating any additional preprocessing steps. The ${\text{LZP}}_{\text{gen}}$ can be calculated for all three graph types. However, it is important to note that the “Naive” graph contains cycles, which makes the interpretation of its ${\text{LZP}}_{\text{gen}}$ less meaningful. Therefore, we recommend that ${\text{LZP}}_{\text{gen}}$ inference be conducted on the “Nucleotide Double Positional” graph when working with nucleotides, and on the “Amino Acid Positional” graph when working with amino acids. These graphs do not contain any cycles and have undergone extensive testing to ensure the stability and accuracy of their ${\text{LZP}}_{\text{gen}}$.

The LZGraph ${\text{LZP}}_{\text{gen}}$ of a given CDR3 Amino Acid/Nucleotide sequence *s* is formally defined as: where *X* is the ordered set of subpatterns obtained by the LZ-76 decomposition (Algorithm 1) applied to sequence *s*. $P(X_{0})$ is the marginal probability of $X_{0}\in \mathrm{Init}$, $w_{i,j}$ is the weight associated to the edge $e_{i,j}$ and $X_{\left|X\right|}\in \text{Term}$. In a scenario where edges exist in a given sequence but were not observed while constructing the LZGraph, the missing values are imputed with the geometric mean of the nonmissing edges of the same sequence. The geometric mean is used due to the probabilities in the LZGraph typically representing a log-linear form. Taking the logarithm of ${\text{LZP}}_{\text{gen}}$ and using the geometric mean for imputing missing values ensures that the resulting imputed values maintain the log-linear interpretation. The geometric mean preserves the multiplicative relationship between the probabilities and ensures that the imputed values align with the overall probability distribution.


$$\begin{array}{c} X=\{x_{1},x_{2},x_{3}\ldots .x_{n}\}\\ P_{\text{gen}}(X)=P(X_{0})\prod_{i=1}^{\left|X\right|}P(X_{i}|X_{i-1})=P(X_{0})\prod_{i=1}^{\left|X\right|}w_{X_{i-1},X_{i}}, \end{array}$$

#### 2.4.2 Graph-based feature vector

There are multiple ways to extract a vector representation of a graph. Here, we used the eigenvector centrality as the feature vector to describe a repertoire. It can be interpreted as the extent to which nodes influence the propagation in the network. The eigenvector centrality (*x*) of a graph is calculated by solving the following equation: where *A* is the adjacency matrix of the graph and λ is the largest eigenvalue of *A*. The eigenvector centrality of node $v_{i}\in V$ is the *i*’th component of *x*, namely $x_{i}$. A node with a high eigenvector centrality value is characterized by being adjacent to other nodes with high eigenvector centrality values.


Ax=λx,

#### 2.4.3 Sequence analysis

We propose two novel approaches for sequence analysis, that facilitate the exploration of gene dynamics at the sequence level and enable comparisons between sequences and repertoires.

1. Sequence variation curve and LZ-Centrality
  The sequence variation curve (Fig. 5A) represents the number of immediate alternatives (out degree) for each LZ-76 subpattern of a given sequence. Given a sequence *S*, it is first decomposed into its LZ-76 subpatterns, denoted as $\hat{S}$. The curve *C* is then defined as:
  $$C=\{\delta^{+}(s_{i})\;|\;\forall s_{i}\in \hat{S}\},$$
  where $\delta^{+}(s_{i})$ is the out degree of node $s_{i}$ based on a given LZGraph.
  We define the average of the *C* curve as the “LZ-Centrality” of a sequence with respect to an LZGraph. This measure serves as a centrality metric, indicating how a sequence relates to the entire repertoire. The higher the LZ-Centrality value is, the more central a sequence is within the graph. This provides a unique perspective on sequence centrality within a repertoire, offering valuable insights into the relationship of a single sequence to many sequences in an efficient manner.
2. Gene variation plot
  The gene variation plots (Fig. 5B–D) depict the number of different V and J genes that are associated with a certain node/edge for a given sequence *S*. It is first decomposed into its LZ-76-subpatterns denoted as $\hat{S}$, for each pair of sequential subpatterns in $\hat{S}$ we extract the observed alleles that are associated with such a transition during the construction of the LZGraph. Alleles that are absent in more than α percent of the sequence path are omitted; the names of the alleles that have been observed through the entire path are colored in red. The process is repeated for both V and J genes separately. The resulting overview (Fig. 5C and D) shows the likelihoods of different alleles for each edge along the sequence path.

### 2.5 Sequence generation

LZGraphs can be used to simulate new sequences with similar statistical properties to the source sequences that were used to construct the graph. Here, we applied two approaches for simulating new sequences, “Unconstrained” and “Genomically Constrained.”

1. Unconstrained method
  The Unconstrained method for sequence generation randomly selects an initial state from the *Init* set based on the empirical probabilities. To the initial subpattern, more nodes are sequentially appended in a stochastic fashion, where the next node is drawn based on the weight distribution of the emanating edges. When a node from the *Term* set is encountered, a conditional stopping procedure is carried out. The conditional stopping procedure checks whether there are any other nodes from the *Term* set that could be reached from the current node. In the case that there are no such nodes, the algorithm halts and uses the encountered node as its terminal state and as the final piece of the generated sequence. On the other hand, if the above does not hold, all the other nodes from *Term* that are reachable from the current node are used to derive a stopping probability $P_{T}$.

$P_{T}(T_{i})$ is defined as the probability of stopping at a given terminal node $\left(T_{i}\in \text{Term}\right)$ conditioned on not stopping at any other terminal state that can be reached from $T_{i}$.
  Formally defined: Let $\Psi (T_{i})$ be the probability of stopping at $T_{i}$ regardless of the initial conditions. That is $\Psi (T_{i})$ equals to the number of paths terminated at $T_{i}$ divided by the total number of paths used to construct the graph. Hence
  $$P_{T}(T_{i})=\frac{\Psi (T_{i})}{\Psi (T_{i})+\sum_{x\in U_{i}}\Psi (x)},$$
  where

$U_{i}=\{\text{all terminal nodes reachable from}\;T_{i}\}\subset \text{Term}.$

2. Genomically constrained
  In the Genomically Constrained method, alongside a random initial state, a random V gene and a random J gene are selected based on the relative frequency observed at the LZGraph’s source repertoire. Similar to the unconstrained method, as long as the generation process of a sequence did not reach a terminal state, the next node is selected randomly based on the relative weight distribution of the out-edges of the current node. Since these out-edges can be associated to any V and J genes, in this method, we take into account only edges that have non zero support for these selected genes. In other words, when building the LZGraph, if we never observed a sequence having the V and J genes that had a transition between the current node and the next node candidate, this edge is excluded from the possible next step.

### 2.6 Comparison with a state-of-the-art model

In this manuscript the comparison will be carried out with “Sonia” (Sethna *et al.* 2020). The Sonia package was developed to infer selection pressures on features of amino acid CDR3 sequences. The inference is based on maximizing the likelihood of observing a selected data sample given a representative preselected sample. The comparison with Sonia was performed using the python open-source library sonia version 0.1.2. The “SoniaLeftposRightpos” model was fitted using the “human_T_beta” chain type, and the repertoire of choice as the “data_seqs” argument of the model. About 200K sequences were generated from the fitted model and were used to further fine-tune it using the “add_generated_seqs” method followed by 30 epochs of “infer_selection” converging on the best fit. For the generated sequence comparison, the library’s “SequenceGeneration” wrapper was used to generate postsequences via the “generate_sequences_post” method. For $P_{\text{gen}}$ comparison the library’s “EvaluateModel” wrapper was used to evaluate a set of sequences, and the “ppost” (Post $P_{\text{gen}}$) result was used for the comparisons.

## 3 Results

### 3.1 LZ-76 BOW vectors to encode repertoires

#### 3.1.1 The LZ-76 BOW dictionary generated from a repertoire encapsulates valuable information

To investigate the effectiveness of using a dictionary generated from applying LZ-76 to a repertoire as a BOW feature vector representation, we conducted the following experiment. We began by creating a unique LZ-76 subpattern dictionary, known as the BOW dictionary, using all relevant repertoires listed in Table 1 for each dataset. Next, we encoded all repertoires using the dataset-specific precalculated LZ-76 BOW dictionary. The repertoire BOW vectors were then normalized based on the repertoire depth. Subsequently, the encoded repertoires (BOW vectors) were projected onto $R^{2}$ using the UMAP algorithm (McInnes *et al.* 2018). This procedure was repeated for each dataset listed in Table 1. Finally, we examined the spatial patterns in the embedded 2D space of the repertoire projections (Supplementary Fig. S5) to identify any noticeable patterns that may exist. Although dictionary sizes and the distribution of frequencies over the LZ-76 subpatterns slightly vary between the three datasets (see Supplementary Fig. S5B), the overall projection in all three cases is robust to the choice of dictionary. A projection of the repertoires from all datasets is shown in Supplementary Fig. S5A, where the LZ-76 BOW vector is constructed using the dictionary based on subpatterns observed in DS1+DS2. Projections using dictionaries from the other datasets are shown in Supplementary Fig. S1. Even though there are subpatterns unique to DS3 and DS4 that do not appear in DS1+DS2 (see Supplementary Fig. S5), by encoding the repertoires using the DS1+DS2 dictionary only, we observe clear segregation between the sets of projected repertoires (most likely due to batch effects; Supplementary Fig. S5). Furthermore, if we consider that DS4 is composed of a few smaller datasets, it is interesting to see that; nevertheless, their mapping into the 2D space is similar (see Supplementary Fig. S1). Alongside projecting the data onto $R^{2}$, we trained a simple decision tree classifier to validate the batch effect hypothesis. We used three different dictionaries derived for each one of the datasets, DS1+DS2, DS3, and DS4. We classified the different sets using such a model, with an average micro F1 score of 0.89±0.02 on 5 folds of cross-validation.

#### 3.1.2 LZ-76 BOW vectors are robust features for classification tasks

**Figure 3.**
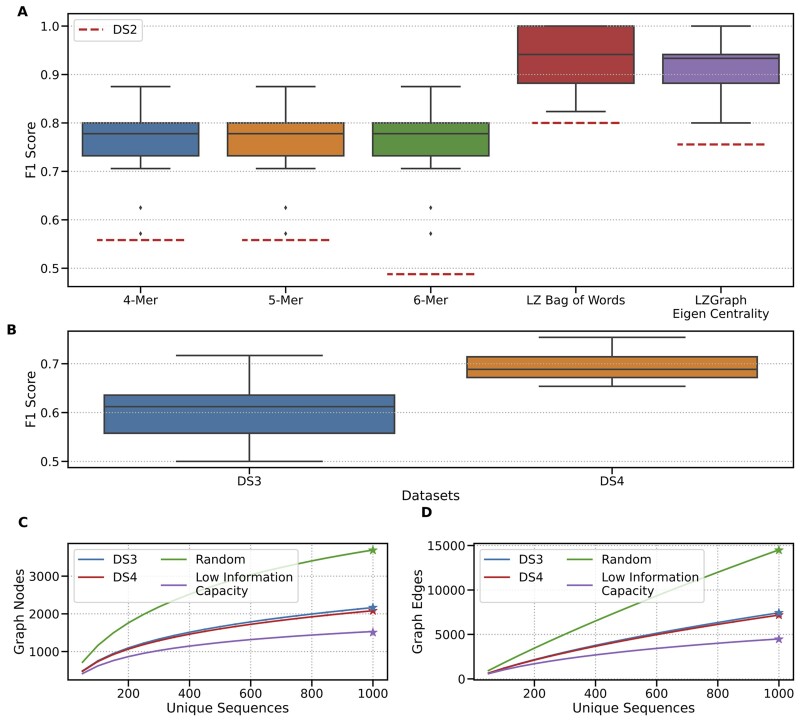
The resulting F1-scores of multiple predictions made using LZ-76 and K-mer features. F1 scores of a 15-fold cross-validation on an AdaBoost model predicting the sample’s sex. (A) features derived from five different extraction methods are represented by the colored boxes. The dashed red line represents the F1 score resulting from the prediction on DS2, using the model trained on DS1. (B) F1 scores of 15-fold cross-validation on a test set of 300 samples out of 950 samples from DS4 and 230 samples out of 785 samples from DS3. (C  and D) Comparison of the K1000 index for repertoires with equal depth and alpha diversity. The blue curve represents unique sequences from DS3, the red curve represents unique sequences from DS4, and the purple curve represents a synthetic repertoire with the same depth and alpha diversity. Notably, the purple curve is derived by generating sequences from a small graph constructed from 2000 unique sequences that maintain the desired alpha diversity and depth. The green curve represents randomly generated sequences of nucleotides, matching the depth and alpha diversity of the other repertoires. The purple and green curves serve as bounds, representing highly diverse repertoires where all sequences are random (green curve) or where the repertoire is based on a limited set of sequences (purple curve). Each curve provides insights into the K1000 index for the corresponding repertoire.

After showing that LZ-76 BOW vectors can be used to identify phenomena such as batch effects between datasets, we tested the applicability of this approach for classifying females and males, in four distinct datasets. We first compared LZ-76 BOW feature vectors to K-mer based vectors. For this, we used DS1 to train an AdaBoost model and compared the resulting F1 scores via 15 folds of cross-validation to rule out any batch effect (see Fig. 3A). The robustness of the BOW feature vectors was then evaluated by introducing a second dataset to the already trained model, namely DS2, while ignoring any new subpatterns that might have appeared while encoding it. Along with surpassing the traditional K-mer approach, the LZ-76 BOW feature vector exhibited enhanced robustness, as evidenced by its notably higher F1 score (dashed red line in Fig. 3A). Notably, in terms of runtime complexity, both the derivation of LZ subpatterns and K-mers are equivalent and bounded by O(N*L), where *N* is the number of sequences in a repertoire, and *L* is the maximum sequence length, resulting in linear time complexity.

### 3.2 Encapsulating sequential relationships via “LZGraphs” results in an “all-in-one” sequence analysis model

While the “naive” LZ-76 BOW representation adequately encapsulated sufficient information, it overlooked a crucial aspect: the sequential relationships among the LZ-76 subpatterns. The naive setting does not impose any constraint on the subpatterns, in fact, the naive setting is no more than normalized count vectors (BOW). By transitioning to a graph (LZGraph), we reattain the sequential relationship between the subpatterns as well as the probabilities associated with those transitions. In this study, we proposed and evaluated three variants of the LZGraph: “naïve,” “Nucleotide Double Positional,” and “Amino Acid Positional” (Fig. 2). These representations offer numerous possibilities for diverse applications, a few of which are highlighted below.

#### 3.2.1 The number of nodes and edges in an LZGraph scale as a power-law with the number of unique source sequences used

The number of unique TCRs in the human body is enormous ($∼10^{10-11}$; Lythe *et al.* 2016). Nevertheless, it constitutes only a tiny fraction of the space of theoretically possible receptors. If we assume that the average CDR3 amino acid length is ∼15 (Ou *et al.* 2018) and ignore the finite number of deterministic patterns that can appear at the beginning and end of the sequence (resulting from the V(D)J recombination process), the theoretical maximum number of sequences would be $20^{15}∼3.3\cdot 10^{19}$.

To measure the connection between the size of an LZGraph and the number of empirical sequences that were used to construct it, we took the following approach. For each dataset, we incrementally added repertoires and constructed from the accumulated set of sequences a “Nucleotide Double Positional” LZGraph (see Section 2). We repeated the above procedure 10 times, shuffling the order of added repertoires.

Supplementary Figure S4 shows the relationships between the number of sequences used for the construction of the graph for DS1+DS2, DS3, and DS4 and the number of nodes (panels A and B. Edges are shown in D and E). It is clear that even for 785 repertoires (DS3) containing 150M sequences, the two curves are not saturating (Supplementary Fig. S4A and B). Plotting these relations in a log–log plot, we observe power-law dependencies. In particular, the number of nodes scales with the number of sequences with a power-law exponent of 0.25–0.28, depending on the dataset, and the number of edges with an exponent of 0.43–0.51, depending on the dataset (Supplementary Table S1). The differences between the datasets may be linked to differences in the diversities in these datasets, but in a nonstraightforward way as the respective Hill diversity curves (Hill 1973) of the samples in these datasets are entangled. These power-law relationships demonstrate that individual repertoires carry a wealth of unique patterns that can add new information, even in cases of graphs that were built from thousands of repertories.

It is interesting to note that according to Turán’s theorem, the maximum number of edges in a directed graph composed of *N* nodes is $N^{2}/4$. As seen in Supplementary Table S1, indeed the exponent of the nodes is slightly more than one half of the exponent of the edges. However, the moderate differences in the ratios between the exponents of the edges and those of the nodes might indicate nontrivial differences in repertoire diversity measures.

#### 3.2.2 LZGraph-based repertoire diversity index

A fundamental task in T cell receptor repertoire sequencing analysis, or more generally, adaptive immune receptor repertoire sequencing (AIRR-seq) analysis involves diversity estimation. Commonly, one first assigns sequences into clones, and then estimates the clonal abundance distribution with a measure of alpha diversity. These measures are borrowed from ecology and include (species) richness, Simpson’s index, Shannon’s entropy, or a continuum of measures suggested by Hill (1973). As such, these measures characterize the distribution of reads attributed to each unique sequence, not taking into account the (dis)similarity between the sequences in a repertoire. Here, we harness the LZGraph data structure and suggest a new measure of repertoire diversity, which goes beyond the clonal abundance distribution. Specifically, we start with the set of unique sequences (which are identical to clones in T cell repertoires) present in a repertoire. We then repeatedly sample this set (without replacement) and construct an LZGraph from each sample. This process is visualized in Fig. 3, and new diversity measures can be defined from it. We show here the use of K1000, defined as the number of nodes in an LZGraph constructed from 1000 randomly sampled unique sequences averaged over 50 sampling procedures. As seen in Fig. 3, the differences between DS3 and DS4 are maintained in a similar fashion to what we saw in Supplementary Fig. S4. Moreover, the empirical K1000 values of DS3 and DS4 are lower than totally random sequences but higher than those of sequences that were generated from an LZGraph built from 2000 unique sequences only.

Since K1000 is calculated from randomly drawn unique sequences, it is not influenced by the clonal abundance distribution. K1000 values are indicative of the information capacity of the corresponding LZGraph, and as such, reflect another type of diversity information. For example, two repertoires can have the same number of unique sequences and the distribution of frequency over these unique sequences. This will result in similar values of alpha diversity but with different K1000 values (“information capacity,” see Fig. 3). An example of K1000 in repertoire comparison can be seen in the Supplementary Fig. S6.

#### 3.2.3 LZGraph-based feature vectors are robust features for classification tasks

Similar to the experiment, we conducted with the LZ-76 BOW feature vector, we tested the capability of the eigen-centrality values of a Naive LZGraph to function as a feature vector by stratifying females and males in four distinct datasets. Here as well, we observed high mean F1 score on 15 folds of cross-validation and robustness when introducing an unseen dataset (Fig. 3A).

#### 3.2.4 Assessing generation probabilities with LZGraphs

An LZGraph stores the information about the relative probabilities to go from one node to a following node that is directly connected to it. These probabilities can yield a generation probability for each sequence ($P_{\text{gen}}$), by multiplying the values attributed to each edge along the path associated with the sequence.

**Figure 4.**
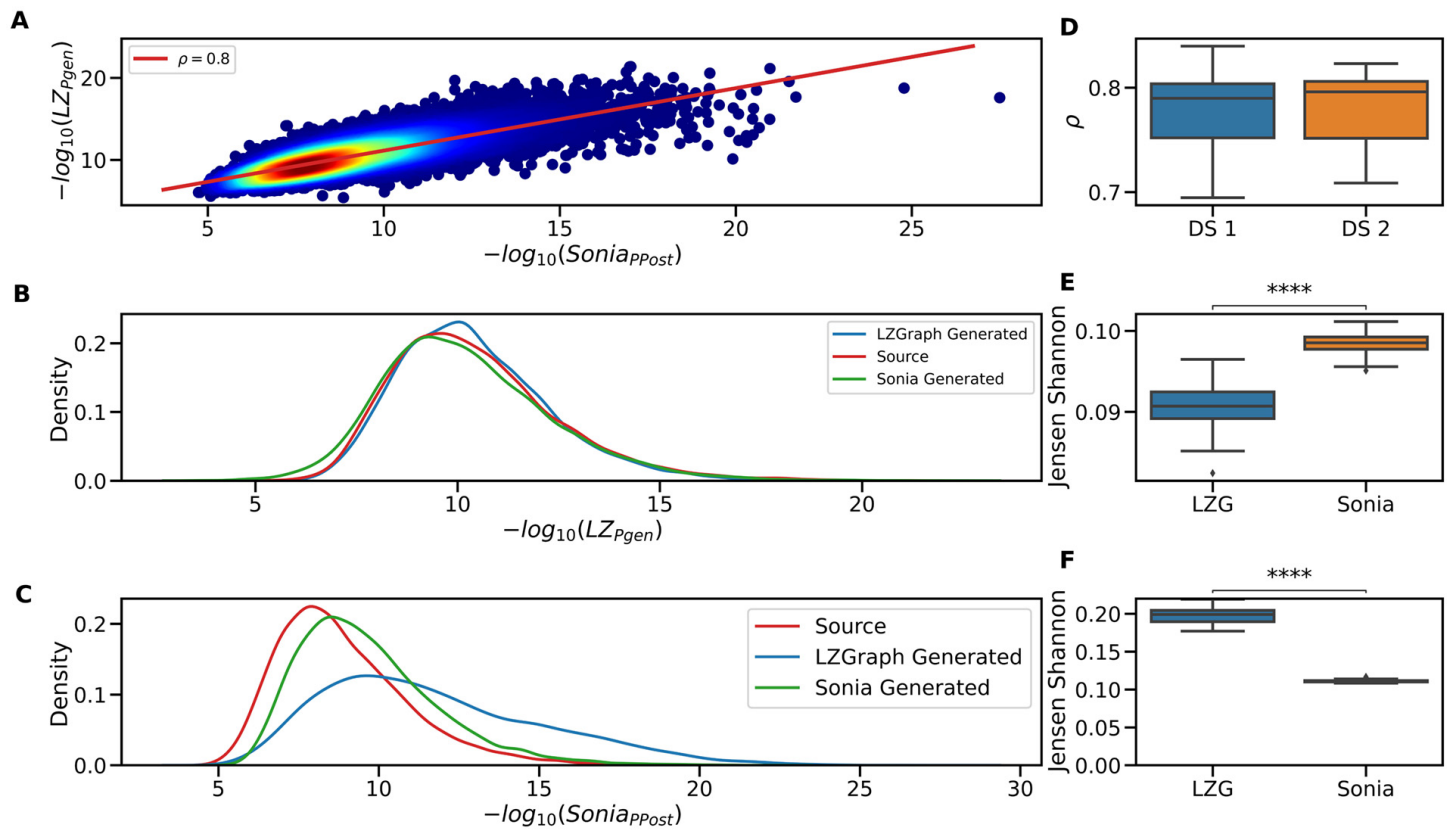
Assessment of LZGraph sequence generation and ${\text{LZP}}_{\text{gen}}$ quality. (A) depicts the correlation between the −log10 ${\text{SoniaP}}_{\text{gen}}$, obtained from SoniaLeftposRightpos, and the −log10 ${\text{LZP}}_{\text{gen}}$, obtained from the “Amino Acid Positional” LZGraph, for an arbitrary repertoire from DS1. The red line represents the linear fit with a Pearson’s correlation coefficient of 0.8 and a slope of 0.76. (B) presents the distribution of LZGraph ${\text{LZP}}_{\text{gen}}$ values for sequences generated via SoniaLeftposRightpos model after fitting it to the source repertoire, as well as the distribution of LZGraph-generated sequences compared to the source repertoire. (D) showcases the Pearson’s correlation coefficient between all sample ${\text{LZP}}_{\text{gen}}$ values and ${\text{SoniaP}}_{\text{gen}}$ values in both DS1 and DS2, calculated using the “Amino Acid Positional” LZGraph and the SoniaLeftposRightpos model, respectively, for a total of 231 compared repertoires. (E) examines the construction of an “Amino Acid Positional” LZGraph and a SoniaLeftposRightpos model for all samples in DS2 (39 in total). An equal number of sequences was generated for each repertoire, and the LZGraph ${\text{LZP}}_{\text{gen}}$ was calculated for sequences generated by both models. The distribution of ${\text{LZP}}_{\text{gen}}$ values in both sets of generated sequences was compared to the distribution of ${\text{LZP}}_{\text{gen}}$ values derived from the source repertoire. The Jensen–Shannon divergence between the generated and source sequences is depicted for each model separately. ****P≤1.00e−04 using Welch’s *t*-test. (F) Jensen–Shannon distance between the Sonia Post Pgen distributions of the source repertoire used to construct an LZGraph and the Sonia LeftPosRightPos model on sequences generated from both models. (C) The same sample as used in panel (B) was taken, the panel shows the distributions of Sonia Post Pgen on the source sequences, generated sequences using Sonia and LZGraph generated sequences.

To validate the $P_{\text{gen}}$s generated via the “Amino Acid Positional” LZGraph, we compared the $P_{\text{gen}}$s produced by it to $P_{\text{gen}}$s produced by “Sonia’s” LeftPosRightPos model (Sethna *et al.* 2020). For this, we calculated both LZGraph’s and Sonia’s “$P_{\text{gen}}$s” for each sequence in a randomly chosen repertoire from DS1. We observed a high correlation between the two $P_{\text{gen}}$ sets (see Fig. 4A). The deviations between the two seem to stem from Sonia’s tendency to attribute extremely low $P_{\text{gen}}$ values in some cases.

It is important to note that in contrast to Sonia, ${\text{LZP}}_{\text{gen}}$ does not require gene annotations and is only based on the sequential relationships between LZ-76 subpatterns in the graph.

#### 3.2.5 *In silico* generation of sequences using an LZGraph

In addition to estimating generation probabilities with an LZGraph, we can leverage the same information stored in the graph to generate artificial sequences. The simulated sequences are expected to have identical statistical properties as the source repertoire used to construct the LZGraph. To evaluate the quality of the sequences generated using the “Amino Acid Positional” LZGraph (in the “Genetic Constrained” mode; see Section 2), we selected an arbitrary repertoire from DS1 and constructed an LZGraph corresponding to that repertoire. Independently, we fitted a Sonia model to the same repertoire, and then generated N sequences from both models, where N is the depth of the original repertoire (Fig. 4A). LZGraph $P_{\text{gen}}$s were calculated for the source repertoire and the two sets of generated sequences, as can be seen in Fig. 4B. When assessing the Jensen–Shannon distance between the generated sequences and the source repertoire, we observe that the LZGraph generated data are closer to the source repertoire in terms of LZGraph generation probabilities. We repeated the same experiment for all repertoires in DS2, and saw identical trends of high correlation between Sonia $P_{\text{gen}}$ and LZGraph $P_{\text{gen}}$ (Fig. 4C), with sequences generated using LZGraph having a lower Jensen–Shannon distance from the source repertoire as apposed to the Sonia generated sequences (Fig. 4D). A comparative analysis was conducted to assess the difference between the source repertoire and the Sonia-generated sequences, using the Jensen–Shannon distance with Sonia post $P_{\text{gen}}$ instead of ${\text{LZG}}_{\text{Pgen}}$. The results indicate that Sonia demonstrates a higher proximity to the source repertoire compared to the LZGraph-generated sequences (Supplementary Fig. S3).

In summary, both methods to generate artificial sequences and to estimate generation probabilities (LZGraph and Sonia) are consistent when assessing the generation probabilities of the sequences generated by the same method. On the other hand, there is a slight difference when assessing the generation probabilities of sequences generated by the other method. The main advantage of the suggested approach (LZGraph) is that there is no need for further annotations to calculate $P_{\text{gen}}$s and to generate artificial sequences that follow the statistical properties of a given repertoire.

### 3.3 Repertoire-specific LZGraph can be used for advanced analyses of individual sequences

The presented approach reveals a plethora of new ways to explore AIRR-seq data. We show below three examples of new ways to analyze with an LZGraph individual sequence in the context of a specific repertoire.

#### 3.3.1 Assessment of sequence centrality

**Figure 5.**
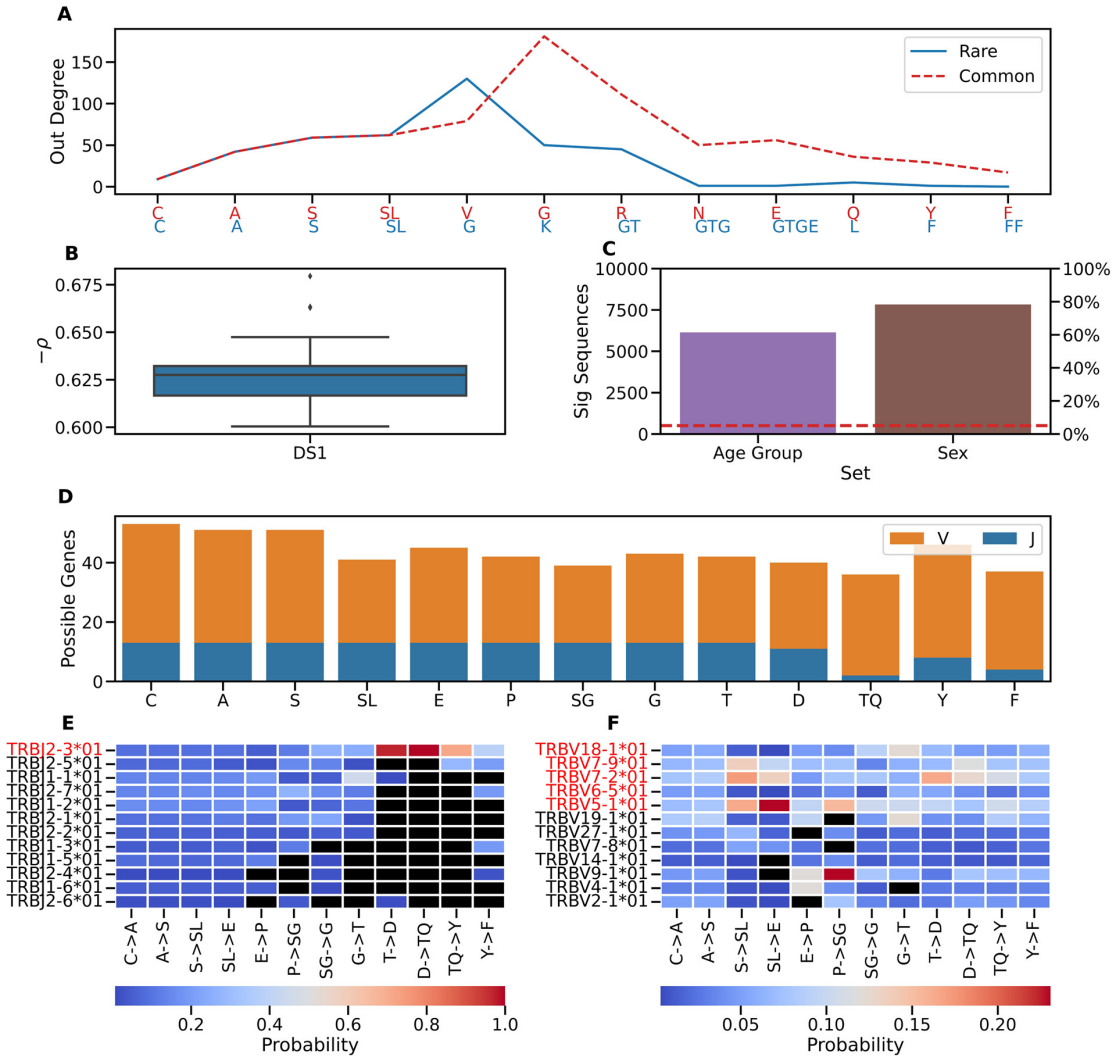
Examples of analysis methods for individual sequences using an LZGraph. (A) Illustrates a curve for two sequences where the curve’s value at each subpattern represents the number of immediately reachable nodes (out-degree) in an LZGraph. The sequences are classified as ’rare’ (blue) and ’common’ (dashed red). (B) Presents the distribution of Pearson correlation values between the average out-degree and the pairwise mean Levenshtein distance. (C) Enumerates the number of sequences with significant *P*-values out of 10 000, resulting from two comparisons performed on the mean out-degree with respect to sexes and age groups across 500 COVID repertoires. Red dashed lines represent the 5% significance level. (D) Displays the count of unique V and J genes that could be utilized at each subpattern position. (E,  F) The heat maps depict all the unique V and J alleles associated with each edge of a sequence. The color scale at each cell indicates the probability of selecting the gene at that edge. A black cube signifies that an allele was never observed at that edge, while a red-colored allele name indicates its consistent observation throughout the entire sequence path.

An LZGraph is structured with regions of differing densities, where each region’s density is a reflection of the support it garners from the sequences used for its construction. As such the LZGraph can be employed to evaluate the path corresponding to a particular sequence. As an illustration, we selected two sequences from a random repertoire from DS1. The LZGraph was constructed using all sequences in the repertoire, and two sequences were chosen based on their *mean* pairwise Levenshtein distance (Miller *et al.* 2009) from all other sequences. The first sequence, labeled “common,” had a significantly lower *mean* Levenshtein distance (8) than the second sequence, labeled “rare” (14). This indicates that the “common” sequence is, on average, more similar to the rest of the repertoire than the “rare” sequence. We then mapped the number of potential alternatives (out-degree) at each subpattern (node) along the sequence for both sequences (Fig. 5A). We term this measure (mean out degree along the nodes of a given sequence, see Section 2) as LZ-Centrality. The “rare” sequence, as determined by the mean Levenshtein distance to all other sequences, has lower out degrees along the sequence compared to the “common” one. Indeed, the mean Levenshtein distance and the LZ-Centrality value of a sequence are correlated (Pearson’s correlations of −0.63±0.015; Fig. 5C). It is worth noting that LZ-Centrality is not correlated only with the *mean* Levenshtein distance (across all sequences in a repertoire), but also with other *mean* pairwise distance metrics, such as cosine similarity, LCS, and Jaro (See example in Supplementary Fig. S2A–D). It is important to mention that the primary objective of the LZ-Centrality measure is not to propose a substitute for the Levenshtein distance, but to underscore the centrality of a sequence within a repertoire as represented by an LZGraph. In Fig. 5D, we demonstrate the applicability of LZ-Centrality by creating 500 LZGraphs for 500 COVID repertoires. We compared the LZ-Centrality values of randomly sampled 10 000 sequences from a single COVID repertoire with respect to other COVID repertoires associated with different age groups and sexes. In 6000 out of these 10 000 sequences, the LZ-Centrality for repertoires associated with different age groups differs significantly (*P*-values lower than 0.05) according to an ANOVA test. Moreover, the LZ-Centrality for repertoires associated with different sexes was significant (*P*-value lower than 0.05) according to a two-sample Weltch’s *t*-test in 7000 sequences. The expected number of sequences with *P*-value lower than 0.05 under a null hypothesis of equal LZ-Centrality mean values between the groups is 500 only. The large numbers that were obtained from these comparisons (6000 and 7000) demonstrate the utility of LZ-Centrality in capturing important biological features. An example of the aforementioned LZ-Centrality differences for a single sequence is shown in Supplementary Fig. S2E and F. LZ-Centrality calculation is highly efficient in terms of runtime and space complexity. Constructing the LZGraph for all sequences in a repertoire costs O(N⋅L), where *N* is the number of sequences in the graph and *L* is the maximum sequence length. This is followed by O(*L*) for the calculation of the mean out degree along a given sequence, resulting in a total inference time of O(N⋅L). In contrast, calculating the pairwise distance metrics typically costs O($N^{2}\cdot L^{2}$). It is worth emphasizing that an analogue procedure to the one presented in Fig. 5D but for other pairwise distance metrics would require orders of magnitude more time, compared to the constant query time of LZ-Centrality calculation.

#### 3.3.2 An LZGraph can store information about flanking genomic regions

An important piece of information when analyzing TCR sequencing data is the identity of the regions that flank the CDR3, specifically the TRBV and TRBJ alleles. These allele annotations can be seamlessly integrated into an LZGraph during its construction process. Notably, to the extent of our knowledge, there is currently no existing tool available to provide insights on amino acid CDR3 sequences in the context of flanking alleles. However, this valuable inference setting becomes accessible without additional effort since the flanked TRBV and TRBJ alleles are automatically incorporated into the graph during its construction, allowing for comprehensive analysis and exploration of the relationships between subpatterns (nodes), transitions (edges), and the associated flanking alleles.

Figure 5B shows an example of what can be learned from such annotated LZGraph on a particular sequence (taken unbiasedly from DS1). As almost all TCRB CDR3 amino acid sequences start with “CAS,” we see that these initial patterns can indeed be attributed to all possible TRBV and TRBJ alleles. On the contrary, the “TQ” subpattern of this sequence for example, has only two possible J alleles and 36 possible V alleles. Such a representation allows us to distinguish between nodes that can appear in all/many flanking alleles and nodes that are allele-specific, from which allele annotations can be inferred.

To gain more information about the sequence shown in Fig. 5B, we applied an analogous logic to the edges as well. From Fig. 5C, we can see that only a single TRBJ allele (TRBJ2-3*01) is consistent with the given sequence, mainly due to two edges with one possible allele (T→D;D→TQ), and from Fig. 5D, we see that five alleles have a nonzero probability to be associated with this CDR3.

### 3.4 LZGraphs performance profile

The importance of algorithmic efficiency in large-scale models such as those presented above is crucial. The methods and data structures presented in this paper were implemented using Python 3.9, and performance profiling was carried out to provide the reader with a summary of the computation time scales for the methods. On top of the fact that the methods presented here do not require a costly preliminary annotation step, they are highly efficient in terms of running times (Supplementary Table S1).

## 4 Discussion

We explore here a new graph-based methodology to analyze TCR repertoires, leveraging the LZ-76 compression. Our model provides the user with a framework for an entire repertoire and single sequences’ analysis in an annotation-free setting. Most of the results presented here do not require germline information such as TRBV/TRBD/TRBJ annotations. Nevertheless, the model can incorporate these annotations, and use them for applications such as synthetic sequence generation and genomic inferences. Furthermore, we introduce a novel diversity index (K1000) that aims to quantify structural patterns, providing a proxy for the potential “information capacity” based on amino acid or nucleotide sequences in an individual’s repertoire. K1000 is conceptually different from common classic diversity measures used today, as it does not rely on unique sequence abundance (see Fig. 3), but rather reflects the “information capacity” of sequences in a given repertoire. It opens the door for new ways to assess repertoire diversity and new comparisons between repertoires.

Throughout the paper, we mainly used an LZGraph to represent the statistical features of a single repertoire. However, it can be used to represent more than one individual. For example, LZGraphs can be constructed for all individuals associated with a particular characteristic (e.g. sex, age, ethnicity, or a particular disease). These resulting graphs can be used to identify features that differentiate between the cohorts and may be harder to trace at the individual level. Moreover, such “master-graphs” can be used to infer ${\text{LZP}}_{\text{gen}}$’s for empirical sequences and to simulate synthetic ones that will better follow the statistical properties of the common denominator of the source cohorts.

There are a few limitations arising from the method due to its specificity to the source repertoire. Naturally, when we construct a probabilistic model based on a single point of reference without any prior, we are limited to the knowledge obtained solely from our observations; thus, two graphs representing two different repertoires will differ in both the nodes and the edges they contain. Naturally, the deeper the repertoire used for the construction of an LZGraph, the more confident the user can be of any difference between two repertoires originating in actual biological differences rather than an effect due to undersampling of the repertoire. Creating a “master-graph” can overcome the depth issue and assess the difference between “master-graphs” rather than between individual repertoires. Another crucial consideration related to repertoire depth is its impact on the K1000 diversity index in our methodology. It is important to note that when the repertoire consists of fewer than 1000 unique sequences, it becomes impossible to calculate the K1000 index directly. In such cases, users are advised to employ an alternative approach by selecting the closest available number of unique sequences to 1000 from their shallow repertoire. This adaptation allows for the estimation of diversity within the given limitations of the dataset. It is important to acknowledge the biochemical interpretation that model-based approaches, like Sonia, offer compared to our sequence-based approach. While Sonia models the actual biochemical process, our methodology focuses primarily on the sequence level. As a result, Sonia provides additional insights and interpretations based on the underlying biochemical mechanisms, which our approach may not directly capture. However, our sequence-based analysis still allows for valuable interpretation of repertoire characteristics based solely on sequence structure, providing complementary perspectives in understanding immune repertoires. The methodology proposed in this paper uncovers an untapped source of feature extraction based on data and string compression algorithms. Although we used the classic LZ-76 algorithm as the compression algorithm in this paper, it is of high interest to explore other compression algorithms for this purpose in future research. In addition to different compression algorithms, it is interesting to explore whether the genomic information stored in the graph captures information that can be used to infer the germline alleles adjacent to the CDR3 region, namely, the TRBV and TRBJ alleles. While the results presented here are based on TCR CDR3 regions, an analogous methodology can be applied to other types of sequences as well. Specifically, it will be interesting to adapt the presented method to BCRs, and explore how somatic hypermutations affect the analysis results. It might even reveal new insights about the somatic hypermutation mechanism.

Notably, our analysis utilizing features derived from the LZGraph (Fig. 3A and B) successfully classified TCR repertoires based on sex. This finding represents a novel and significant contribution to the field, as to our knowledge, no previous studies have demonstrated such a classification capability for TCR repertoires.

In summary, this paper takes the first step in representing TCR repertoire data using lossless compression algorithms. Future work is needed to explore the many possible extensions and adaptations of the presented approach in the context of AIRR-seq and other types of biological sequences.

In conclusion, this study pioneers the use of lossless compression algorithms in the representation of TCR repertoire data, laying the groundwork for a transformative approach in AIRR-seq and beyond. As we venture forward, the potential adaptations and enhancements of this innovative approach promise to unlock unprecedented insights in the world of biological sequencing and enhance our understanding of immune repertoires.

## Supplementary Material

btad426_Supplementary_Data
