# Supplementary material for: A novel approach to T-cell receptor beta chain (TCRB) repertoire encoding using lossless string compression
Source: Bioinformatics. 2023 Jul 7;39(7):btad426. doi: 10.1093/bioinformatics/btad426 (PMC10348835; doi:10.1093/bioinformatics/btad426)
Supplement: btad426_Supplementary_Data [file btad426_supplementary_data.pdf]

Supporting Information for

A Novel Approach to T-Cell Receptor Beta Chain (TCRB) Repertoire Encoding Using Lossless String Compression

Thomas Konstantinovsky, Gur Yaari

This PDF file includes:

Figs. S1 to S5
Table S1

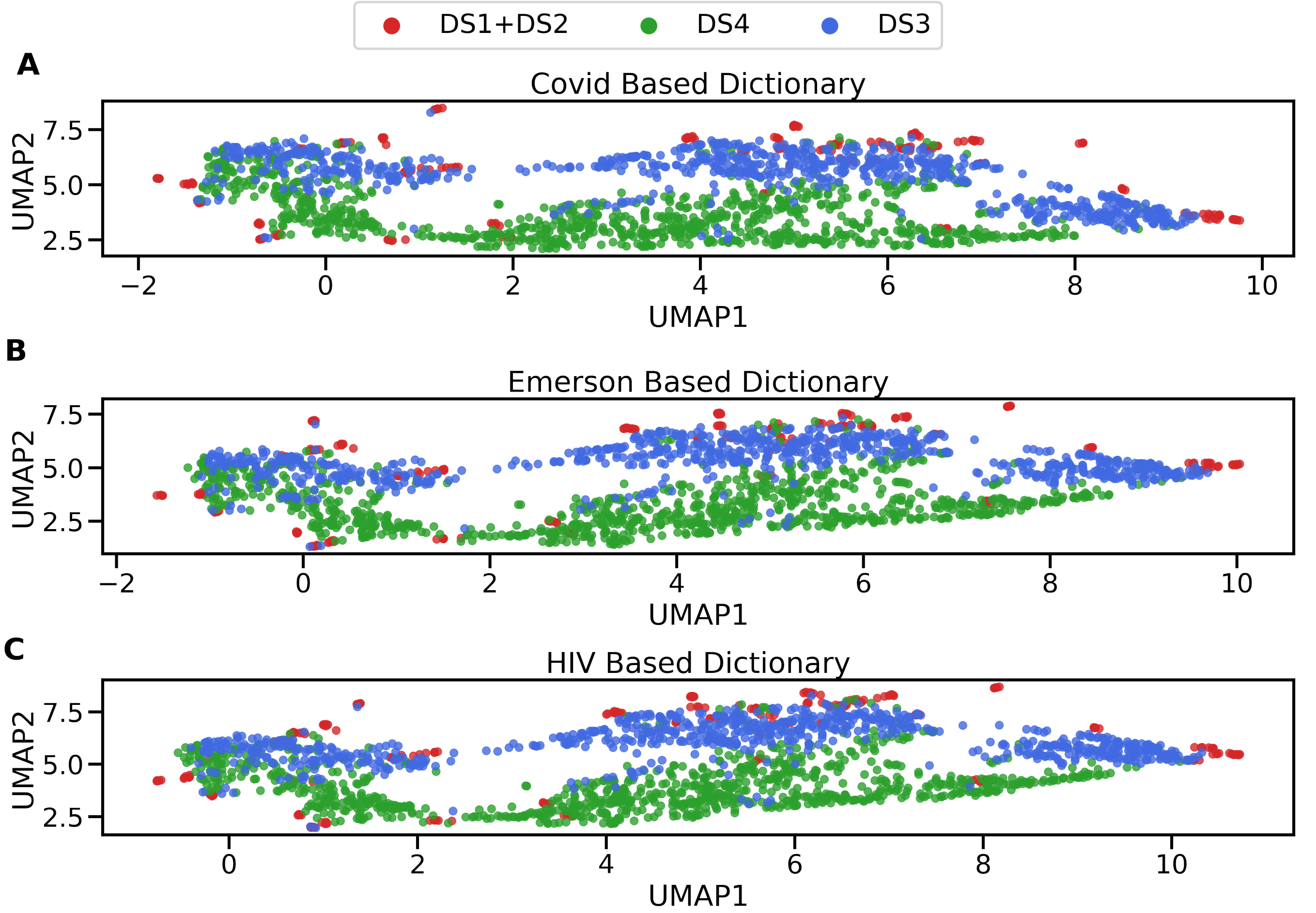

Fig. S1. Projections onto R^2 using UMAP of the datasets used in the paper. At each panel the datasets were encoded using a BOW dictionary derived from only one dataset. The dictionaries in panels (A-C) are DS1+DS2,DS3 and DS4, respectively.

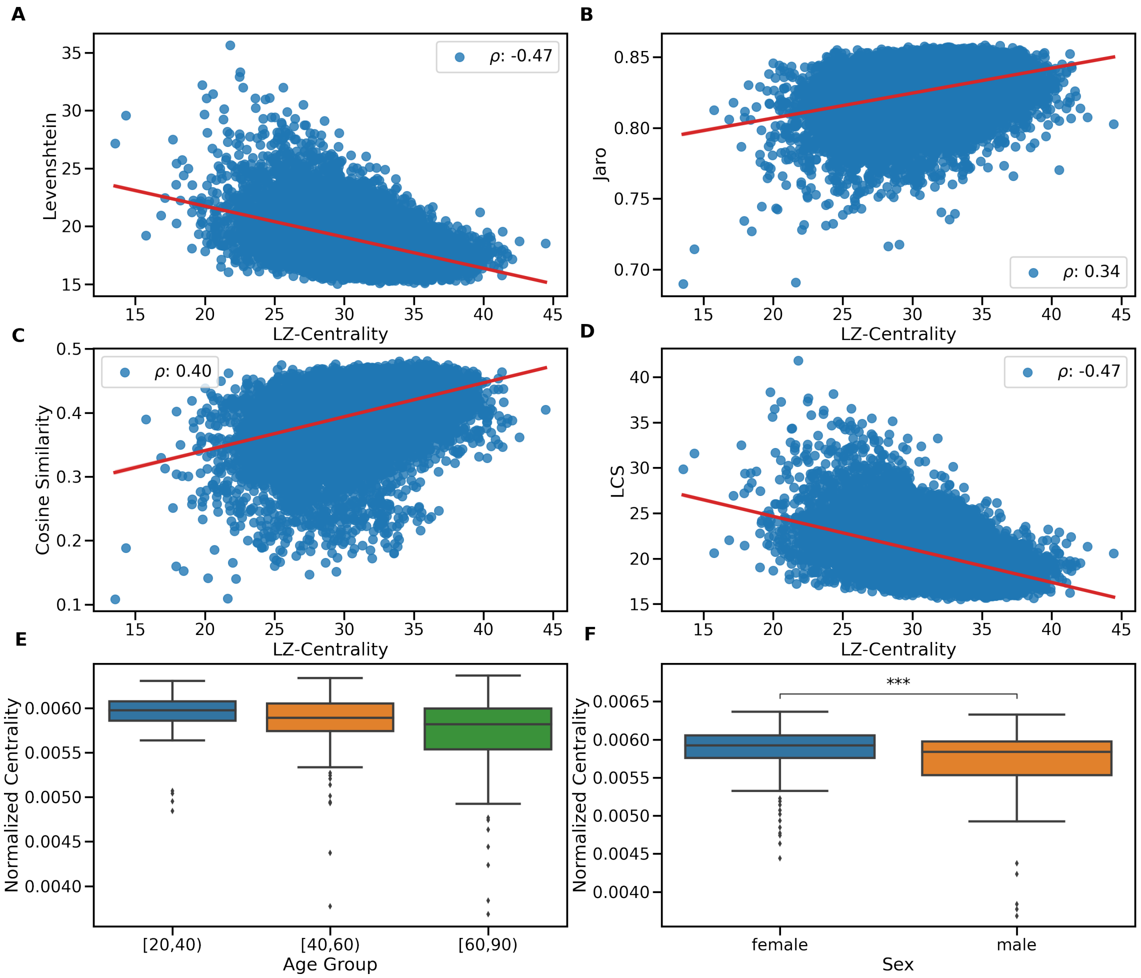

Fig. S2. (A-D) The relationship between four mean pairwise distance metrics (Levenshtein, Cosine Similarity, Jaro, and LCS) and the Mean Out Degree generated by the LZGraph for a given sequence. The plot shows sequences (blue dots) from an arbitrary repertoire extracted from DS2 dataset Next the LZ-Centrality of the following sequence: "TGTGCTAGTGGTTTGGCAGGGGGCAATGAGCAGTTCTTC" was calculated over 500 repertoires. **(E)** Shows the normalized centrality distribution of a particular sequence for different age groups of 500 COVID repertoires. The normalized Centrality was calculated for a randomly selected sequence from a COVID repertoire using 500 LZGraphs, each corresponding to a unique COVID repertoire. The normalized centrality values resulting from this procedure were categorized into Age groups based on the metadata of the 500 repertoires. An ANOVA test was conducted on the normalized centrality values, revealing a statistically significant p-value < 0.001. **(F)** Similar to panel E, the same procedure was followed, but this time a Welch's T-test was performed to assess the difference in normalized centrality between Sex categories. The analysis yielded a significant p-value < 10-e-3.

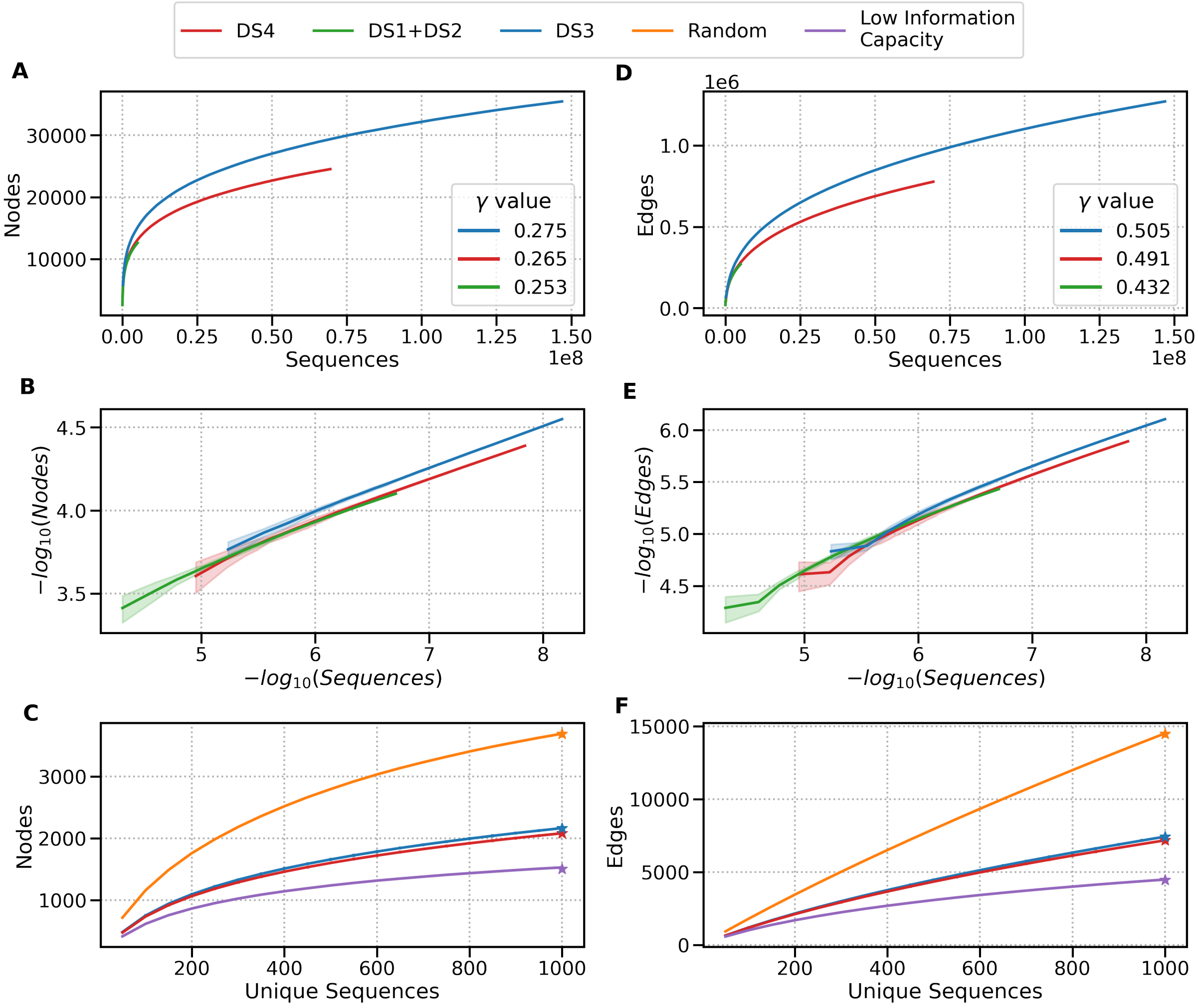

Fig. S3. LZGraph Graph Nodes and Edges Growth Rate as a Basis for K1000 Diversity Index. (**A+B**) show the growth in the number of graph nodes based on the number of sequences used in both regular and log-log scales. γ is the power law coefficient, namely, $f(x) = a \cdot x^{-\gamma}$ (**D+E**) shows the growth in the number of graph edges based on the number of sequences used in both regular and log-log scales. (**C+F**) show 4 examples of the K1000 index,the orange curve represents the K1000 index for randomly generated sequences. The blue and red curves represent 30 samples each for DS3 and DS4, respectively. The Purple curve represents the K1000 index for the same number of unique sequences as in all other examples generated using an LZGraph built from only 2000 unique sequences.

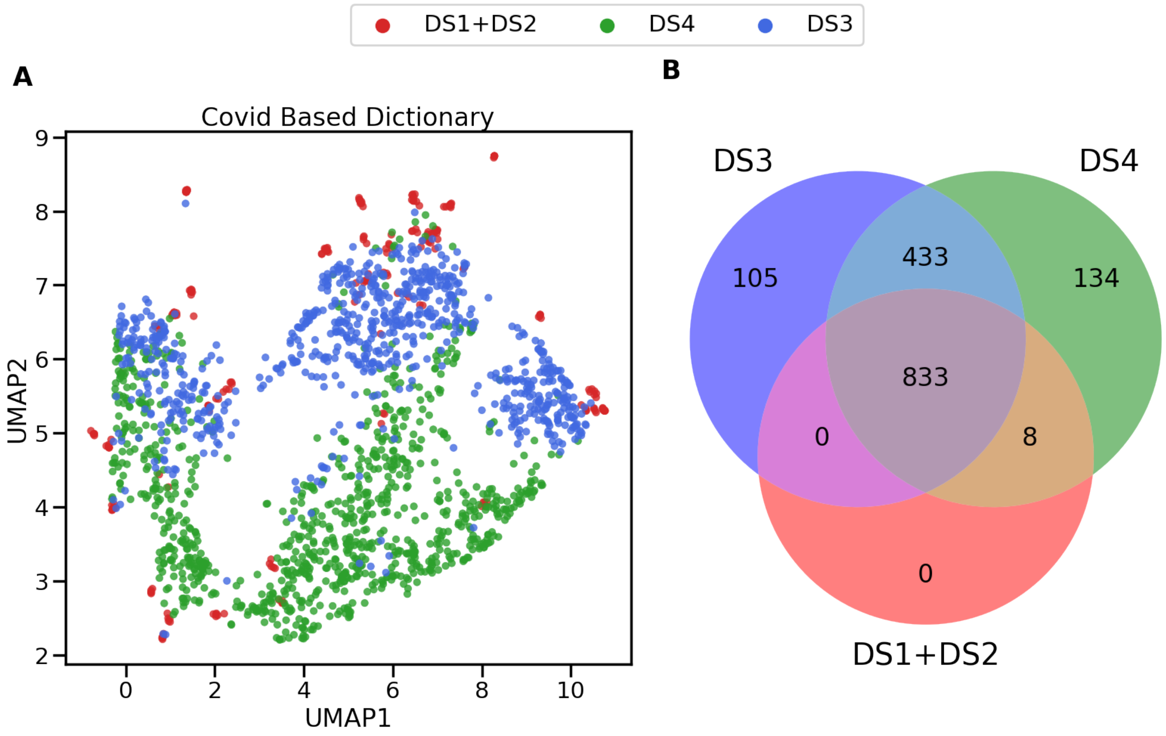

Fig. S4. BOW dictionary encoded repertoires spatial clustering and dictionary similarity. (**A**) All four datasets were projected onto $\mathbb{R}^2$ using UMAP. The features used to project each repertoire were all sub-patterns discovered in DS4. Each color represents a dataset. (**B**) Venn Diagram of unique LZ-76 sub-patterns observed in each dataset.

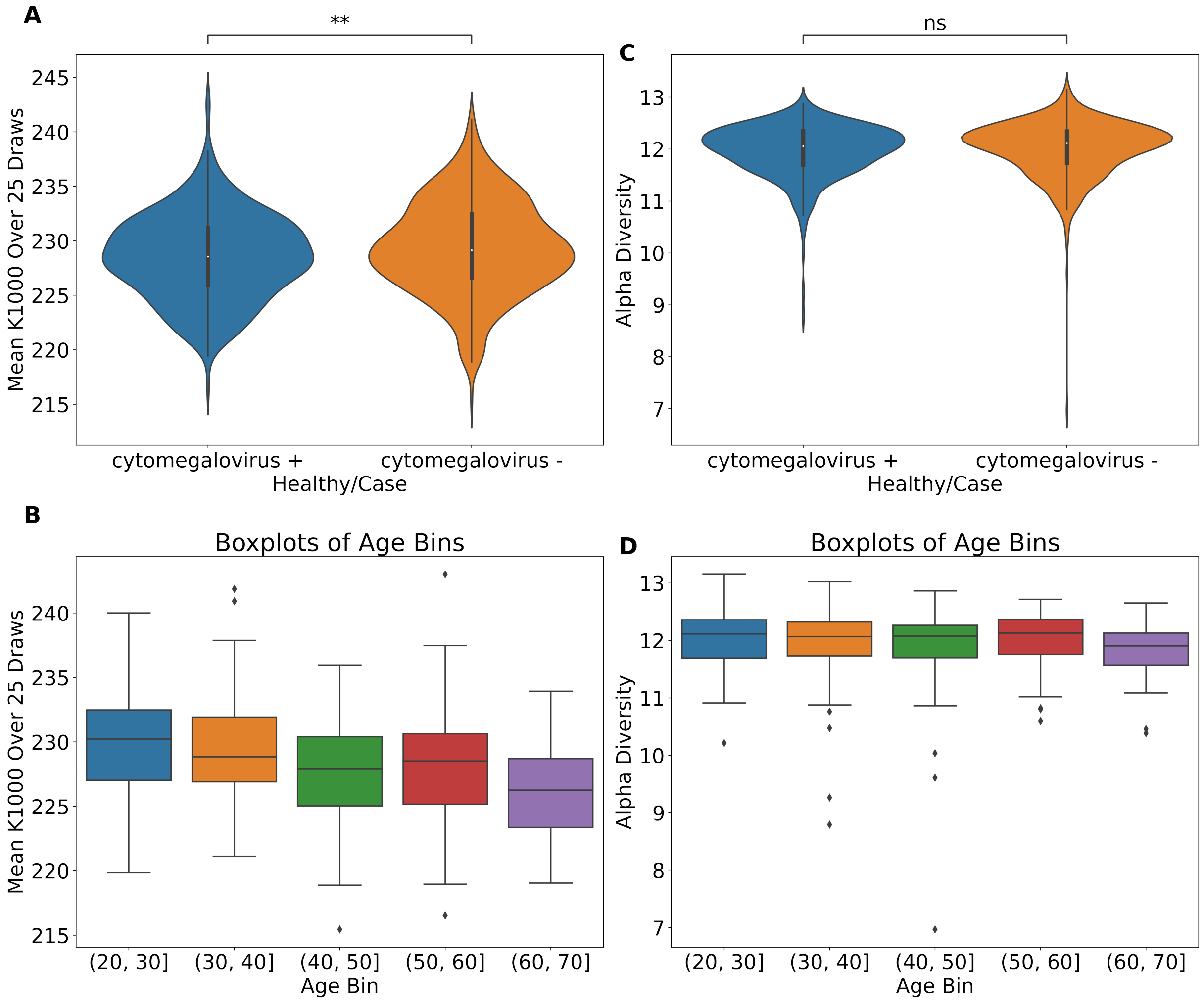

Fig. S5. Difference Between Different Age and Case Repertoires in the Emerson CMV Dataset Based on K1000 (A) Comparison of the distributions of K1000 values between CMV-positive and CMV-negative repertoires. CMV-positive repertoires are shown in blue. The presence of ** indicates a significant difference with a p-value lower than 1.00e-03, determined using a two-sided Welch's t-test. **(B)** Comparison of the distributions of K1000 values among different age groups. An ANOVA test revealed a highly significant difference with a p-value < 1.9e-08. **(C)** The same plot as in (A), but for alpha diversity indexes. A two-sided Welch's t-test resulted in a non-significant p-value (>0.05), indicating no significant difference in alpha diversity between CMV-positive and CMV-negative repertoires. **(D)** The same plot as in (B), but for alpha diversity indexes. An ANOVA test resulted in a non-significant p-value (>0.05), indicating no significant difference in alpha diversity among different age groups

Table S1. A summary of run times of the different models and methods presented in this paper.

Model	Method	Seconds per 1e3 Sequences	Seconds per 1e4 Sequences	Seconds per 1e5 Sequences
Naive	Build	0.09s	0.51s	4.6s
Nucleotide Double Positional		0.33s	1.53s	10.22s
Amino Acid Positional		0.2s	0.74s	4.66s
SoniaLeftposRightpos		12.88s	15.82s	19.30s
Naive	Infer Generation Probability	0.03s	0.34s	3.65s
Nucleotide Double Positional		0.03s	0.32s	3.16s
Amino Acid Positional		0.05s	0.11s	1.1s
SoniaLeftposRightpos		11.35s	41.55s	341.79s
Naive	Sequence Synthesis	0.54s	6s	69s
Nucleotide Double Positional		0.19s	2.67s	33.43s
Amino Acid Positional		0.17s	2.52s	29.08s
SoniaLeftposRightpos		7.39s	7.65s	8.53s
